# The Long-Term Effects of a Peer-Led Sex Education Programme (RIPPLE): A Cluster Randomised Trial in Schools in England

**DOI:** 10.1371/journal.pmed.0050224

**Published:** 2008-11-25

**Authors:** Judith Stephenson, Vicki Strange, Elizabeth Allen, Andrew Copas, Anne Johnson, Chris Bonell, Abdel Babiker, Ann Oakley, the RIPPLE Study Team

**Affiliations:** 1 Department of Infection and Population Health, Division of Population Health, University College London, London, United Kingdom; 2 Social Science Research Unit, Institute of Education, London, United Kingdom; 3 London School of Hygiene & Tropical Medicine, London, United Kingdom; 4 Medical Research Council, Clinical Trials Unit, London, United Kingdom; The University of Adelaide, Australia

## Abstract

**Background:**

Peer-led sex education is widely believed to be an effective approach to reducing unsafe sex among young people, but reliable evidence from long-term studies is lacking. To assess the effectiveness of one form of school-based peer-led sex education in reducing unintended teenage pregnancy, we did a cluster (school) randomised trial with 7 y of follow-up.

**Methods and Findings:**

Twenty-seven representative schools in England, with over 9,000 pupils aged 13–14 y at baseline, took part in the trial. Schools were randomised to either peer-led sex education (intervention) or to continue their usual teacher-led sex education (control). Peer educators, aged 16–17 y, were trained to deliver three 1-h classroom sessions of sex education to 13- to 14-y-old pupils from the same schools. The sessions used participatory learning methods designed to improve the younger pupils' skills in sexual communication and condom use and their knowledge about pregnancy, sexually transmitted infections (STIs), contraception, and local sexual health services. Main outcome measures were abortion and live births by age 20 y, determined by anonymised linkage of girls to routine (statutory) data. Assessment of these outcomes was blind to sex education allocation. The proportion of girls who had one or more abortions before age 20 y was the same in each arm (intervention, 5.0% [95% confidence interval (CI) 4.0%–6.3%]; control, 5.0% [95% CI 4.0%–6.4%]). The odds ratio (OR) adjusted for randomisation strata was 1.07 (95% CI 0.80–1.42, *p* = 0.64, intervention versus control). The proportion of girls with one or more live births by 20.5 y was 7.5% (95% CI 5.9%–9.6%) in the intervention arm and 10.6% (95% CI 6.8%–16.1%) in the control arm, adjusted OR 0.77 (0.51–1.15). Fewer girls in the peer-led arm self-reported a pregnancy by age 18 y (7.2% intervention versus 11.2% control, adjusted OR 0.62 [95% CI 0.42–0.91], weighted for non-response; response rate 61% intervention, 45% control). There were no significant differences for girls or boys in self-reported unprotected first sex, regretted or pressured sex, quality of current sexual relationship, diagnosed sexually transmitted diseases, or ability to identify local sexual health services.

**Conclusion:**

Compared with conventional school sex education at age 13–14 y, this form of peer-led sex education was not associated with change in teenage abortions, but may have led to fewer teenage births and was popular with pupils. It merits consideration within broader teenage pregnancy prevention strategies.

**Trial registration:**

ISRCTN (ISRCTN94255362).

## Abstract 2

Judith Stephenson and colleagues report on a cluster randomized trial in London of school-based peer-led sex education and whether it reduced unintended teenage pregnancy.

## Editors' Summary

**Background.:**

Teenage pregnancies are fraught with problems. Children born to teenage mothers are often underweight, which can affect their long-term health; young mothers have a high risk of poor mental health after the birth; and teenage parents and their children are at increased risk of living in poverty. Little wonder, then, that faced with one of the highest teenage pregnancy rates in Western Europe, the Department of Health in England launched a national Teenage Pregnancy Strategy in 2000 to reduce teenage pregnancies. The main goal of the strategy is to halve the 1998 under-18 pregnancy rate—there were 46.6 pregnancies for every 1,000 young women in this age group in that year—by 2010. Approaches recommended in the strategy to achieve this goal include the provision of effective sexual health advice services for young people, active engagement of health, social, youth support, and other services in the reduction of teenage pregnancies, and the improvement of sex and relationships education (SRE).

**Why Was This Study Done?:**

Although the annual under-18 pregnancy rate in England is falling, it is still very high, and it is extremely unlikely that the main goal of the Teenage Pregnancy Strategy will be achieved. Experts are, therefore, looking for better ways to reduce both teenage pregnancy rates and the high rates of sexual transmitted diseases among teenagers. Many believe that peer-led SRE—the teaching (sharing) of sexual health information, values, and behaviours by people of a similar age or status group—might be a good approach to try. Peers, they suggest, might convey information about sexual health and relationships better than teachers. However, little is known about the long-term effectiveness of peer-led SRE. In this randomized cluster trial, the researchers compare the effects of a peer-led SRE program and teacher-led sex education given to13- to 14-y-old pupils on abortion and live birth numbers among young women up to age 20 y. In a cluster randomized trial, participants are randomly assigned to the interventions being compared in “clusters”; in this trial, each “cluster” is a school.

**What Did the Researchers Do and Find?:**

Twenty-seven schools in England (about 9,000 13- to 14-y-old pupils) participated in the RIPPLE (Randomized Intervention of PuPil-Led sex Education) trial. Each school was randomly assigned to peer-led SRE (the intervention arm) or to existing teacher-led SRE (the control arm). For peer-led SRE, trained 16- to 17-y-old peer educators gave three 1-h SRE sessions to the younger pupils in their schools. These sessions included practice with condoms, role play to improve sexual negotiating skills, and exercises to improve knowledge about sexual health. The researchers then used routine data on abortions and live births to find out how many female study participants had had an unintended pregnancy before the age of 20 y. One in 20 girls in both study arms had had one or more abortions. Slightly more girls in the control arm than in the intervention arm had had live births, but the difference was small and might have occurred by chance. However, significantly more girls in the intervention arm (11.2%) self-reported a pregnancy by age 18 than in the intervention arm (7.2%). There were no differences between the two arms for girls or boys in any other aspect of sexual health, including sexually transmitted diseases.

**What Do These Findings Mean?:**

These findings indicate that the peer-led SRE program used in this trial had no effect on the number of teenage abortions but may have led to slightly fewer live births among the young women in the study. This particular peer-led SRE program was very short so a more extended program might have had a more marked effect on teenage pregnancy rates; this possibility needs to be tested, particularly since the pupils preferred peer-led SRE to teacher-led SRE. Even though peer-led SRE requires more resources than teacher-led SRE, this form of SRE should probably still be considered as part of a broad teenage prevention strategy, suggest the researchers. But, they warn, their findings should also “temper high expectations about the long-term impact of peer-led approaches” on young people's sexual health.

**Additional Information.:**

Please access these Web sites via the online version of this summary at http://dx.doi.org/10.1371/journal.pmed.0050224.

- This study is further discussed in a *PLoS Medicine* Perspective by David Ross
- Every Child Matters, a Web site produced by the UK government, includes information on teenage pregnancy, the Teenage Pregnancy Strategy, and teenage pregnancy statistics in England
- Directgov, an official government Web site for UK citizens, provides advice for parents on talking to children about sex and teenage pregnancyand advice for young people on sexual health and preventing pregnancy
- Teachernet, a UK source of online publications for schools, also provides information for parents about sex and relationships education and the UK government's current guidance on SRE in schools
- Avert, an international AIDS charity, also provides a fact sheet on sex education
- The Sex Education Forum in the UK is the national authority on Sex and Relationships Education

## Introduction

The sexual health of young people in the United Kingdom has been declared a crisis [1]. UK teenage pregnancy rates remain the highest in Western Europe and sexually transmitted infections (STIs) continue to rise [2]. In 2000, the Department of Health in England launched a national strategy to halve teenage (under 18 y) pregnancy rates by 2010 [3]. Improving sex and relationships education (SRE) at school is a key theme of the strategy, and peer-led SRE has been highlighted as a promising approach [4].

The term “peer” refers to people of equal status. Peer-led (sex) education can therefore be defined as “teaching or sharing of (sexual health) information, values and behaviours by members of similar age or status group” [5]. The egalitarian interaction between young people may allow more open and culturally relevant communication about sexual health issues, with peers conveying information in a more credible and appealing way than teachers. Several theories are used to support this view, based on the importance of social networks and the values and beliefs of peers in influencing people's behaviour [6]. However, systematic reviews have shown a lack of reliable evidence for the benefits of peer interventions [7,8]. A meta-analysis of randomised trials of a range of school, clinic, and community-based interventions to reduce teenage pregnancy concluded that such interventions do not delay sex, improve contraceptive use, or reduce pregnancy [9]. But none of the school-based trials involved long-term follow-up, and all relied on self-report measures.

To examine the long-term effects of peer-led SRE on sexual health outcomes, we did a randomised trial of a peer-led SRE programme with follow-up throughout the teenage years (the RIPPLE trial, Randomised Intervention trial of PuPil-Led sex Education). The programme had been used sporadically in schools in England before the trial; it was designed along pragmatic rather than explicitly theoretical lines, with emphasis on generalisability. Planned interim analyses showed that peer-led SRE was more popular than teacher-led SRE and associated with significantly fewer girls reporting sexual intercourse by age 16 y [10]. Here we present the final follow-up to age 20 y, with anonymised linkage of all girls in the trial to routine (statutory) reports of abortions and live births, to assess the effectiveness of peer-led SRE in reducing unintended teenage pregnancy.

## Methods

### Design and Purpose of Trial

Schools were randomised to peer-led SRE (intervention) or to continue their usual teacher-led SRE (control) when pupils were aged 13–14 y (in 1998 and 1999), with follow-up to age 20 y. The trial was designed to compare the effectiveness of the two approaches to reducing abortion, unprotected sexual intercourse, and improving the quality of sexual relationships. The trial design (Text S1 and S2) is described in detail elsewhere [11].

### Participating Schools and Pupils

Eligible schools in central and southern England were comprehensive, from rural and urban areas, with intake of girls and boys to age 18 y. All pupils in Year 9 (8th grade, aged 13–14 y) were eligible to take part unless their parents opted to withdraw them, following written information to parents [11]. The pupils were given oral and written information about the study and the voluntary nature of participation was stressed when they were invited to complete questionnaires. In intervention schools, all pupils in Year 12 (11th grade, aged 16–17 y) were eligible to be peer educators; those wishing to participate did not have to meet any selection criteria. Peer educators gave signed consent to participate. The trial was approved by the committee on the ethics of human research at University College London.

### Intervention and Implementation

The intervention was designed by an external team of health promotion practitioners with experience in delivering peer-led sexual health programmes in schools. It was based on a programme that had been used in a variety of schools in England, and was not designed around any particularly theoretical framework. It was piloted to ensure that it could be implemented in a standardised way across different types of schools [12]. The peer educators were trained to prepare classroom sessions aimed at improving the younger pupils' skills in sexual communication and condom use, and their knowledge about pregnancy, STIs, contraception, and local sexual health services. They delivered three 1 h sessions of SRE to Year 9 pupils, using participatory learning methods and activities focusing on relationships, STIs, and contraception (Box 1). These sessions replaced the usual teacher-led SRE delivered during Personal, Health and Social Education in intervention schools. Teachers were not present in the classroom. Control schools continued with their usual teacher-led SRE and received £1800 to spend on anything except SRE.

### Outcomes

The primary outcome, chosen as a clear indicator of an unintended pregnancy, was abortion before age 20 from routine (statutory) data collected until 31 December 2004. Since the abortion rate by itself cannot reflect all unintended pregnancies, we also obtained routine data on live births (collected until 10 June 2005 and age 20.5 to correspond to the abortion data) to help interpret any difference by arm in the abortion rate. Following list-cleaning of the trial register through the National Health Service (NHS) central register, girls were matched to routine data on live births from two sources: (1) registration of births, using name, date of birth and postcode where available; and (2) registration of maternities, using NHS number. We sent the trial register to the Office for National Statistics for matching to birth registrations, and to Northgate Information Solutions for matching to maternity registrations. Girls were matched to routine data derived from statutory abortion notification forms using date of birth and postcode, with confirmation of matches using name (held on paper records only). We sent the trial register to the Department of Health for abortion matching. For both live births and abortions, matching was done by staff who were blind to allocation, and individually matched data were aggregated and returned to us as a simple count per school, so that no participant with an abortion or live birth could be identified.

Further secondary outcomes based on questionnaire data included self-reported pregnancy and unintended pregnancy; sexual intercourse and use of contraception (at first and last sex); regretted or pressured sex (at first and last sex), quality of relationship with current partner (enjoyment of time together and ease of communication); self-reported STD diagnosed by a doctor or nurse and attendance at a clinic for advice about sex, knowledge of the emergency contraceptive pill, and ability to identify local sexual health services.

### Data Collection

Questionnaires were completed in the classroom at baseline and at approximately 6 and 18 mo after intervention. The third follow-up questionnaire was completed in the classroom by participants still attending school at approximately 54 mo after baseline; participants who had left school were provided with questionnaires by post, by home visit from an interviewer, or failing that, via their general practitioner (GP). The mean (standard deviation) age of students at third questionnaire follow-up was 18.24 (0.65) y (18.15 [0.44] y excluding those obtained by GP follow-up). Process data were gathered from the questionnaires and from extensive observation of peer educator training, sessions of peer-led and teacher-led SRE, focus groups with pupils, and interviews with key staff [13,14].

### Sample Size

The trial was powered to detect a 33% reduction in the cumulative incidence of abortion by age 20 (from expected rate of 9% to 6%, based on routine data for England and Wales in 1993). Taking the cluster design into account, and assuming the coefficient of variation for the primary outcome to be 0.2, the trial would need, in each arm, 14 schools with an average of 150 girls to have 80% statistical power to detect such a reduction at 5% significance. To achieve at least 150 girls aged 13–14 y per school, we recruited two successive cohorts of Year 9 pupils in autumn 1997 and autumn 1998 respectively.

### Randomisation

Before randomisation, schools were divided into high-, medium-, and low-risk strata according to seven risk variables [11]: socioeconomic status (the proportion of pupils having free school meals), ethnicity (the proportion of black and Asian students), educational attainment (the proportion gaining five or more General Certificates of Secondary Education [GCSE]), continuing education (the proportion staying on after age 16), the quality and quantity of pre-existing school sex education, the attitude of the school toward health promotion (the availability of information and links with outside agencies), and local family planning services (convenient location or opening times, youth-friendliness, and level of use by students). From this information, the schools were ranked and divided into three risk strata of approximately equal size. Randomisation of schools occurred within strata, using a computer-generated sequence of allocation of block size ten for each. This process resulted in 15 experimental schools and 14 control schools.

### Statistical Methods

Primary analysis was by intention-to-treat. All female pupils were included in analysis of abortions (primary outcome) and live births ascertained from routine data through anonymised linkage. For 44 (19%) abortions, age at abortion was not available. These abortions were included in analysis by age 20, but not by age 18. Analysis was based on the method of generalised estimating equations (GEE) [15], incorporating the correlation of data within schools, and based on the robust variance estimator.

For outcomes obtained from the third follow-up questionnaire, we present the prevalence of each outcome and ORs with and without weighting. (Where the outcome referred to time until present, questionnaires returned via GP were excluded because this occurred substantially later than other responses). In summary, the weights were designed to deal with the missing data for those pupils who did not complete a third follow-up questionnaire, and are based on how the completion rates are seen to vary by factors collected previously, i.e., according to responses to earlier completed questionnaires. Such weighting is a standard approach to dealing with missing data, particularly in surveys. For example suppose pupils reporting having had sex in an earlier questionnaire are seen to be less likely to complete the third follow-up. In this case those pupils reporting sex earlier who do complete a third follow-up questionnaire will be given more weight in analysis so as to represent themselves and also other similar pupils who did not complete the third follow-up. Specifically we stratified pupils into eight strata according to gender and the questionnaire they last completed (none, baseline, first, or second follow-up). Within each stratum we used the latest questionnaire data (and trial arm and the school risk stratum) to build a logistic regression model for completion of third follow-up questionnaire, using a forward stepwise fitting procedure. Fitted probabilities of questionnaire completion were calculated for each pupil who completed a questionnaire and their inverse was taken as the weight, and then scaled to the total number of pupils within that stratum and trial arm. Pupils in strata where no previous data was collected were assigned a weight of one.

Factors considered for inclusion in the models for questionnaire completion were: previous sexual experience, attitudes to premarital and casual sex, confidence with condom use, ability to say no to something sexual, knowledge of STDs, ability to identify local sexual health services, communication with parents/guardians, religion, attitude to school, housing tenure, and whether parent/guardian are employed. In the final models, a low school risk stratum, the ability to say no, knowledge of STDs, and disapproval of casual and premarital sex increased the completion rate, whereas having had sex and the ability to identify sexual health services reduced the completion rate in most strata.

For outcomes based on questions about first sex asked in each follow-up questionnaire, data were taken from the first questionnaire completed after first sex, after excluding pupils who had first sex before baseline or after age 19 y. These data are not weighted. When analysing these outcomes, we adjusted for age at first sex to remove any confounding from the higher response rate at third follow-up in the intervention arm, but interpret the resulting OR with caution because age at first sex may be itself affected by the intervention. We made further adjustment for pupil-level baseline factors (dislike of school and housing tenure) known to predict a range of outcomes. This replaced adjustment for school risk stratum incorporated in the primary analysis. To provide measures of the effect of the intervention on binary outcomes, the OR is given from logistic regression. For continuous outcomes, the difference between the means in the two arms is used, derived from linear regression. These regressions are performed using the GEE methodology. The weighted analysis described above was done using the survey analysis functions of STATA 7, broadly equivalent to GEE with an independence working correlation. For ordinal outcomes, ORs are given, based on proportional odds logistic regression, and again survey analysis methodology was used to account for the correlation within schools. All *p*-values in the text are from adjusted analyses.

A single outcome—had sex by 18 y—was based on Kaplan-Meier techniques using data from all follow-up questionnaires. This analysis was based on working out an estimate for each school, then working out weighted averages of these 343 potentially eligible schools across schools to provide a figure for each study arm, and the difference between these to assess the effect of the intervention. The figures for each school were weighted by the inverse of the estimated total variability of the cumulative incidence for that school. This estimate of the total variance for a school is the sum of its within-school variance and the between-schools estimated variance, the latter estimated as described elsewhere [11].

## Results

**Figure 1:**
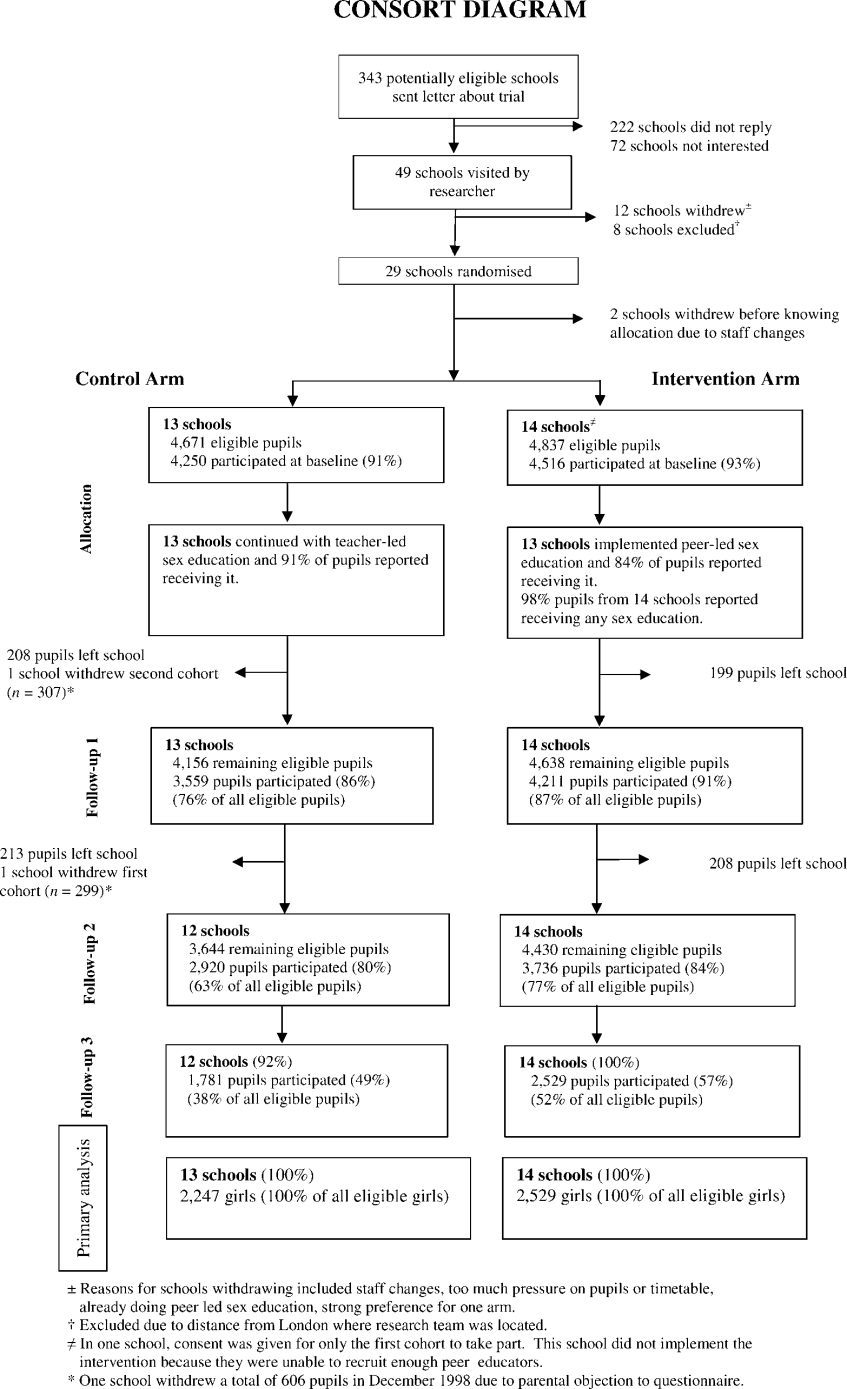
Consort Diagram Flow diagram of participants in the trial.

**Figure 2:**
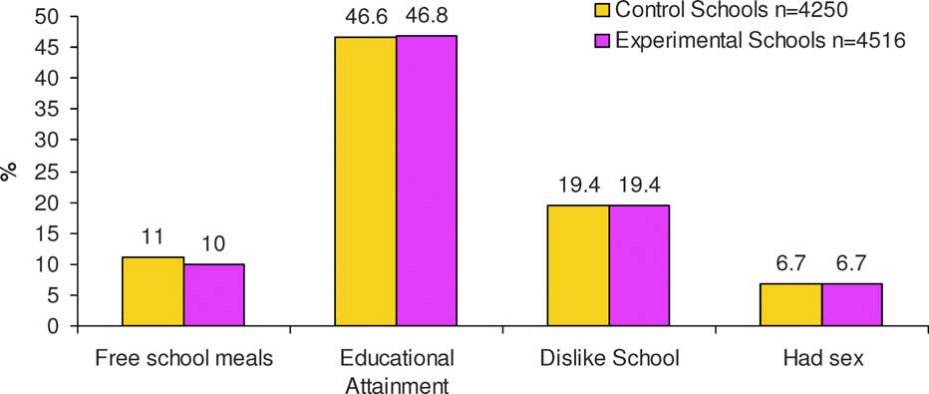
Baseline Data at Mean Age 13.7 Years Proportion of pupils in the schools eligible to free school meals (extracted from Ofsted reports) relates to all pupils on school roll; educational attainment (percentage of pupils obtaining a score of five or more GCSE grades A*–C, taken from DfES performance tables, http://www.dfes.gov.uk) relates to pupils older than participants. “Had sex” refers to heterosexual intercourse.

Parents did not consent for 1.9% of Year 9 pupils (1.5% from control, 2.3% from intervention schools) to take part in the research. Two schools (one from each arm) withdrew due to staff changes without knowing their random allocation (CONSORT diagram [Figure 1]). One intervention school was unable to implement the intervention, but contributed to follow-up. The two arms were well balanced with respect to demographic data and the proportion of pupils reporting sexual intercourse at baseline (Figure 2). For each arm, the proportion of pupils attaining five or more GCSE grades A*–C was similar to that for all state secondary schools in England (mean 46% [16]).

### Evaluation of Processes

Process data showed some variation in the implementation of peer-led SRE across the 14 intervention schools. The intervention school that did not implement peer-led SRE could not recruit enough peer educators. According to interviews with teachers, Year 9 pupils in control schools received a mean of three (range zero to seven) sessions of SRE; 97.5% of students in intervention schools and 91.1% in control schools reported having received some sex education in Year 9 [14,17]. Similar topics were addressed in both types of sex education, but the biggest difference was in skill-based activities such as practising putting a condom on a model (76.4% intervention pupils; 26.8% control, *p* < 0.001). The peer educators tended to be high academic achievers from more advantaged backgrounds than the Year 9 pupils and this, coupled with the formal classroom setting, may have influenced the “peer like” nature of their interactions with pupils [18,19].

### Evaluation of Outcomes

Table 1 shows pregnancy outcomes, determined by linkage to routine data, by trial arm and by school. There was no difference in the estimated cumulative proportion of girls with one or more abortions by age 20 y, which is the primary outcome. For this outcome, we estimated the design effect to be 1.13 by comparing the standard errors for the log-odds for the intervention effect adjusted for randomisation strata, with and without acknowledgment of the clustering by school. With an average school size of 170 girls, the design effect corresponds to an intracluster correlation coefficient (ICC) of only 0.0008. This ICC was substantially reduced by the stratification; without this adjustment the ICC was 0.0034. The proportion of girls who had one or more live births was lower in the peer-led arm, although not significantly. The number of girls with live births resulting from the matching process (*n* = 415) was consistent with the expected total number of births (450, based on data for girls of the same age residing in the same areas as the participating schools), while the number of girls having an abortion (*n* = 232) was 77% of the expected number of abortions (300, based on the proportion [40%] of under 20 conceptions that end in abortion nationally). The lower rate for abortions probably reflects missing postcode for 25% of girls (28% control, 21% intervention, *p* = 0.21) and missing date of birth for 3.2% of girls (4.2% control, 2.1% intervention, *p* = 0.01). The proportion of girls with missing NHS number for live birth matching was 3.3% in control schools and 1.8% in intervention schools (*p* = 0.01).

**Table 1:**
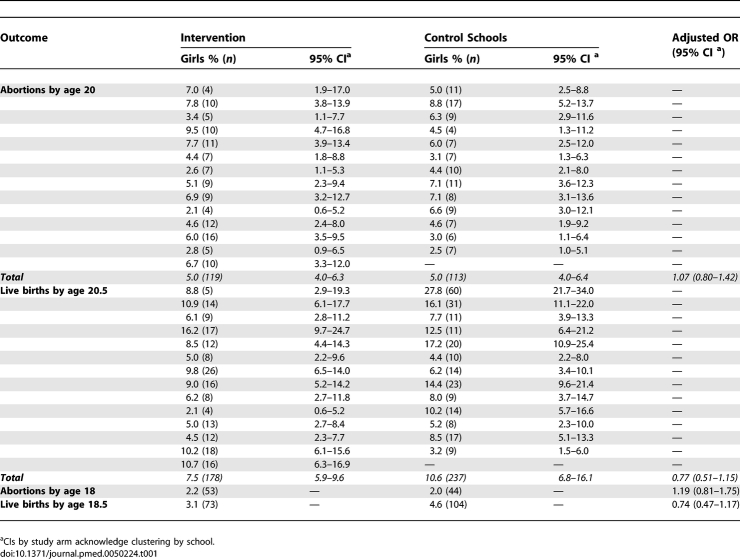
Abortions and Live Births from Matching to Routine Data, by School

Questionnaires at age 18 y were completed by significantly more (*p* = 0.001) intervention pupils (52.3% overall; 61.3% girls, 43.7% boys) than control pupils (38.1% overall; 45.4% girls, 31.4% boys). Of these, 3.1% of intervention pupils and 4.4% of control pupils completed questionnaires via GP follow-up (*p* = 0.024). The proportion of girls who had had sex by age 18.0 y was not significantly different between peer-led and control arms [72.4% versus 73.2% for girls (estimated difference −0.3, 95% confidence interval [CI] −4.4 to 3.8) and 56.8% versus 58.2% for boys (−2.4, 95% CI −7.2 to 2.3)]. Weighted analysis showed significantly fewer self-reported pregnancies among girls in the peer-led arm by age 18 (Table 2). Girls, but not boys, in the peer-led arm were more likely to report using contraception at last sex, although the significance of the difference was not retained in weighted analysis (Tables 2 and 3). There were no significant differences between arms for girls or boys in unprotected first sex, regretted or pressured sex (at first and last sex), quality of relationship with current partner, diagnosed STD, knowledge of the emergency contraceptive pill, or ability to identify local sexual health services.

**Table 2:**
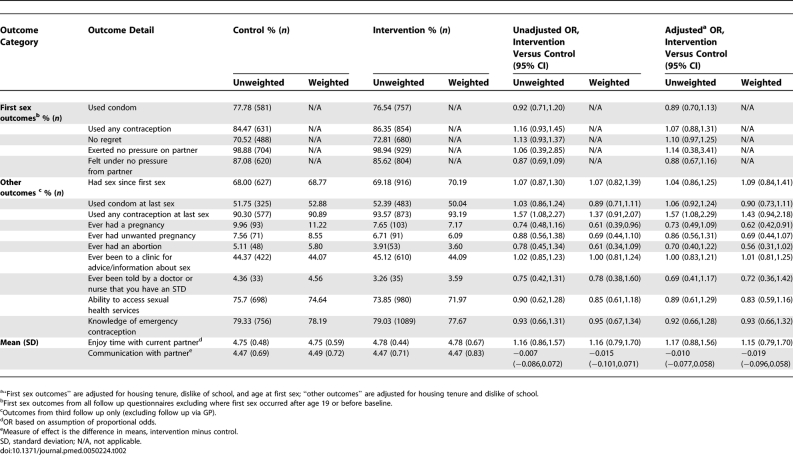
Secondary Outcome Measures for Girls

**Table 3:**
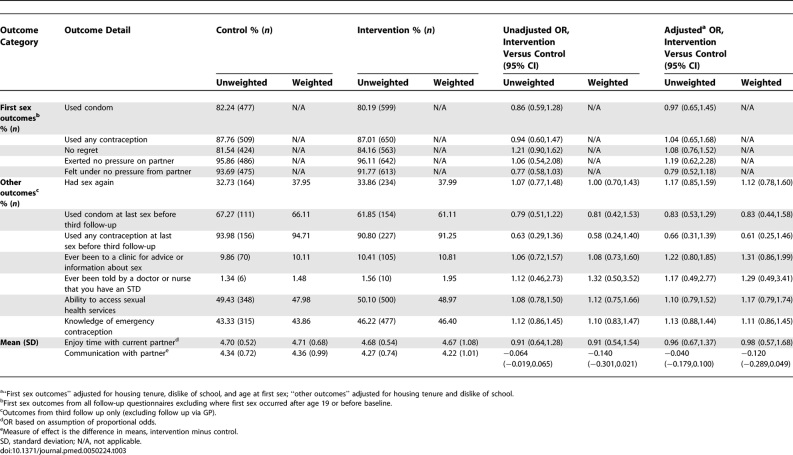
Secondary Outcome Measures for Boys

**Figure 3:**
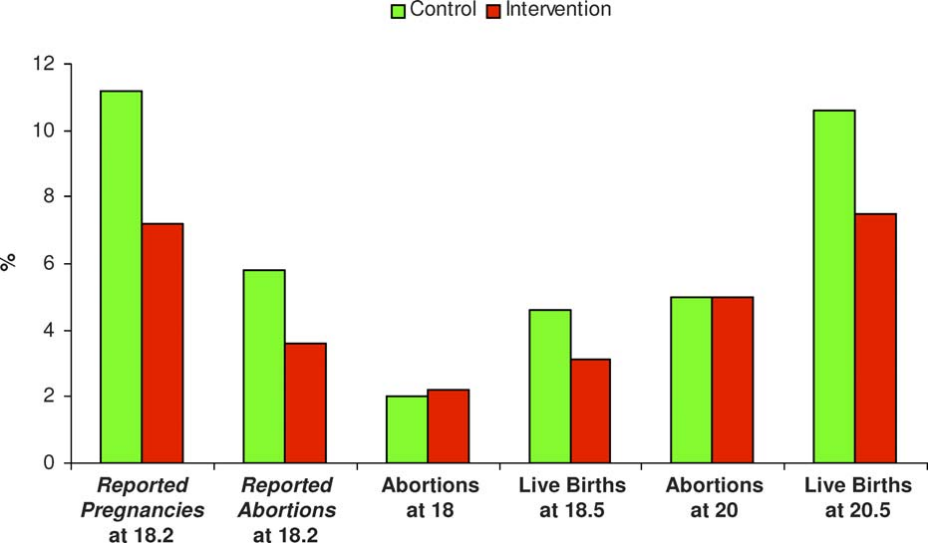
Pregnancy Outcomes at Follow-Up Data on self-reported conceptions and abortions at mean age 18.2 y are from weighted responses to third follow-up questionnaire; abortions at ages 18 and 20, and live births at ages 18.5 and 20.5, are from routine (statutory) data.

Figure 3 summarises data from Tables 1 and 2 on pregnancy, abortion, and live birth, by different methods of ascertainment. Data on reported pregnancy and matched live births are consistent in being lower in the peer-led arm. Data on matched abortions show no difference between arms, while self-reported abortion is lower in the peer-led arm.

## Discussion

We did a cluster randomised trial to compare the long term effects of a brief programme of peer-led SRE (intervention arm) with conventional teacher-led SRE (control arm) on sexual behaviour and pregnancy outcomes in over 9,000 pupils (aged 13–14 y at baseline) from representative schools in England. By age 20 y, the proportion of girls having one or more abortions was the same (5%) in each arm of the trial. Other outcome data showed that the peer-led programme was more popular with pupils and may have led to fewer live births to teenage girls.

The model of peer-led SRE used in this study was designed to be generalisable to a wide variety of schools. The intervention was brief, essential input of teachers was kept to a minimum, and the training and support of peer educators was acceptable to school staff and pupils. The students in the two groups of schools were very similar at baseline, and there is no evidence that contamination occurred between arms or that any school engaged in unexpected forms of SRE. Similar topics (e.g., contraception and reproduction) were addressed in both types of SRE, but the nature of the interaction between peers and pupils was clearly different to that between teachers and pupils [18,19].

Since the trial did not have a “no sex education” control group, we cannot draw conclusions about the full impact of sex education, only about the effect of different approaches in schools. A similar trial in Scotland compared conventional school sex education with a theoretically based, specially designed programme delivered by teachers, and found no impact on conception or abortion rates [20]. It concluded that the potential for classroom teacher-delivered sex education to influence young people's behaviour might already have been reached by current school provision. More effective programmes may have to address socioeconomic divisions in society and the importance of parental influence.

Discovering which interventions work best to reduce teenage pregnancy is challenging: a meta-analysis of randomised trials of various school, clinic, and community-based interventions in North America concluded that they had not delayed sex, improved contraceptive use, or reduced pregnancies [9], while a systematic review that included nonrandomised studies reached more positive conclusions [21]. RIPPLE aimed to provide more reliable information about the effectiveness of school sex education. As far as we are aware, it is the only randomised trial of peer-led sex education to include blinded ascertainment of pregnancy outcomes from routine (statutory) data, as well as self-reports. To interpret these data together, it is important to consider the strengths and limitations of the study in relation to ascertainment of outcomes. Blinding of participants to type of sex education was not possible, but matching to routine sources was blinded. The process of obtaining informed consent from pupils was unlikely to result in differential accuracy between arms in reporting pregnancy outcomes, but the response rate to the final questionnaire was lower in the control arm (which we think is partly due to greater engagement of school staff in follow-up procedures in the intervention arm), and pupils at higher risk of pregnancy are likely to be harder to follow-up. We attempted to address this problem by weighting the results according to differential follow-up rates, but weighting can only take account of risk factors for non-response measured in the trial, i.e., based on the responses given in earlier completed questionnaires.

For example, if girls who have had a live birth since their last completed questionnaire are less likely to be followed up because they have dropped out of school due to motherhood, then weighting is unlikely to fully remove the resulting bias in the estimation of self-reported pregnancy. Matching participants to routinely collected data offers the major advantage of eliminating bias due to reporting inaccuracy or loss to follow-up, being dependent only on the quality and completeness of recorded information on the trial register and on birth registration or abortion notification forms. The number of live births resulting from the matching process was closer to the expected number than the number of abortions was, reflecting more missing data in the trial register for abortion matching (postcode and date of birth) than for live birth matching (NHS number only). Any bias from undermatching of abortions is likely to be toward underestimation of abortion in the control arm, since the control arm had more missing data than the intervention arm. Finally, the self-reported pregnancy data may have been more reliable than the self-reported abortion data, because the question wording was simpler (“Have you ever been pregnant?”) and there were fewer missing responses than for the question on abortion (“If yes [to ever being pregnant], did you decide to have a termination or abortion?”).

In summary, the matched abortion data, showing no difference between intervention and control schools, are probably more reliable than the self-reported data showing fewer abortions in the peer-led arm. Data on matched live births and reported pregnancy are more consistently lower in the peer-led arm, suggesting the possibility that the reduction in live births is a real, rather than chance, finding (Figure 3). Differences in other outcomes were small but tended to favour peer-led SRE, both in terms of pupil satisfaction (reported at age 16 [10]) and sexual risk behaviour.

What are the implications of these trial findings for peer-led sexual health interventions more generally? They suggest that the long-term benefits of some peer-led interventions are not as evident as their popularity. Delivering SRE that pupils find more satisfactory is clearly a positive outcome, but this needs to be balanced against the considerable demands that implementation of peer-led SRE places on schools and their staff. This difficulty may have contributed to the low school participation rate in RIPPLE, although some schools were put off by the research requirements that would not apply to routine implementation. The programme we evaluated was brief (three 1 h sessions) but typical of the time allocated to SRE in schools in England. With more resources we might have evaluated a longer programme, but we cannot know whether that would have had more impact. The relation between intervention duration and impact on sexual health is not straightforward; in the SHARE study, a 20-session adult-led SRE programme that was robustly designed and evaluated in Scotland had no impact on conception, abortion, or sexual behaviour [20].

Concern about the economic, health, and social costs of teenage pregnancy, to both individuals and governments, has led countries such as the United Kingdom and United States to make concerted efforts to reduce their high rates. Under-18 conceptions fell by 27% in the USA between 1990 and 2000 and by 12% in England between 1998 and 2005 [22,23]. Possible explanations include favourable economic trends, better sexual health education, delayed first sex, and more use of long-acting contraceptive methods—although the relative impact of these factors is much disputed [24,25]. Strong socioeconomic, religious, and cultural influences underlie teenage pregnancy rates and provide the context in which sex education and other interventions aim to inform, support, and protect young people. Pupils still complain that most SRE is delivered too late and is too “biological” (i.e., technical). According to pupils in this study, the features of good SRE include use of active teaching methods, receiving key information that is relevant to their needs, an opportunity to practice skills and a teacher or educator with relevant expertise and respect for pupils who holds similar values about sex, uses familiar language, is not moralistic, and can make the sessions fun. These features occurred in both types of SRE in this trial, although more frequently in the peer-led programme [26].

The strongest government lever for improving sex education in the UK is to make SRE mandatory within Personal Social and Health Education, as several European countries have done for decades. Evidence is growing that good sex education delays the onset of sexual activity [21] rather than hastening it, as feared by some parents and fuelled by some media reports. On the basis of interim results, the RIPPLE intervention was included in a short list of SRE programmes to help accelerate progress through the Teenage Pregnancy Strategy in England [27]. Taken as a whole, the results of this trial support consideration of the RIPPLE programme as part of a much broader strategy to reduce teenage pregnancy. They may also temper high expectations about the long-term impact of peer-led approaches to improving young people's sexual health.

## Supporting Information

Text S1 CONSORT Checklist (63 KB DOC)

Text S2 Study Protocol (2.4 MB DOC)

## Abbreviations

GCSE: General Certificates of Secondary Education
GEE: generalised estimating equations
GP: general practitioner
ICC: intracluster correlation coefficient
NHS: National Health Service
OR: odds ratio
SRE: sex and relationships education
STI: sexually transmitted infection
