# Supplementary material for: The Long-Term Effects of a Peer-Led Sex Education Programme (RIPPLE): A Cluster Randomised Trial in Schools in England
Source: PLoS Med. 2008 Nov 25;5(11):e224. doi: 10.1371/journal.pmed.0050224 (PMC2586352; doi:10.1371/journal.pmed.0050224)
Supplement: Text S1 — (63 KB DOC) [file pmed.0050224.sd001.doc]

# CONSORT Statement 2001 - Checklist
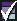


**Items to include when reporting a randomized trial**

| ***PAPER SECTION* And topic** | Item | **Descriptor** | **Reported on**  **Page #** |
| --- | --- | --- | --- |
| TITLE & ABSTRACT | 1 | [How participants were allocated to interventions](http://www.consort-statement.org/index.aspx?o=1107) . Schools were randomly allocated to the experimental or control group. | 1 & 2 |
| *INTRODUCTION* Background | 2 | [Scientific background and explanation of rationale](http://www.consort-statement.org/index.aspx?o=1016). The poor sexual health of young people is a major concern. Peer-led sex education is widely believed to be effective in reducing unsafe sex, but reliable evidence from long-term studies is lacking. | 4 |
| *METHODS* Participants | 3 | [Eligibility criteria for participants](http://www.consort-statement.org/index.aspx?o=1017#3a) and the [settings and locations where the data were collected](http://www.consort-statement.org/index.aspx?o=1017#3b). Eligible schools in central and southern England were comprehensive, with intake of girls and boys to age 18. | 6 |
| Interventions | 4 | [Precise details of the interventions intended for each group and how and when they were actually administered](http://www.consort-statement.org/index.aspx?o=1021). The experimental intervention, peer-led sex and relationships education (SRE), consisted of 3 classroom sessions using participatory learning methods and activities focusing on relationships, contraception and STI. Sessions were delivered to 13-14 year olds by trained peers aged 16-17 years from the same school. Control schools continued with their usual teacher-led SRE. | 2 & 6 |
| Objectives | 5 | [Specific objectives and hypotheses](http://www.consort-statement.org/index.aspx?o=1022). To assess the effectiveness of one form of school-based peer-led SRE in reducing unintended teenage pregnancy and improving sexual health | 2 & 6 |
| Outcomes | 6 | [Clearly defined primary and secondary outcome measures](http://www.consort-statement.org/index.aspx?o=1023#6a) and, when applicable, any [methods used to enhance the quality of measurements](http://www.consort-statement.org/index.aspx?o=1023#6b) (*e.g.*, multiple observations, training of assessors). Primary outcome: abortion before age 20 using routine data. Secondary outcomes: pregnancy and unintended pregnancy, sexual intercourse and use of contraception, regretted or pressured sex, quality of relationship with current partner, diagnosed STD etc | 7 & 8 |
| Sample size | 7 | [How sample size was determined](http://www.consort-statement.org/index.aspx?o=1024#7a) and, when applicable, [explanation of any interim analyses and stopping rules](http://www.consort-statement.org/index.aspx?o=1024#7b). Sample size powered to detect a 33% reduction in abortion by age 20 | 8 |
| Randomization -- Sequence generation | 8 | [Method used to generate the random allocation sequence, including details of any restrictions](http://www.consort-statement.org/index.aspx?o=1025) (*e.g*., blocking, stratification) Computer generated allocation sequence of block size 10 for three school strata (high, medium, low risk schools) | 9 |
| Randomization -- Allocation concealment | 9 | [Method used to implement the random allocation sequence](http://www.consort-statement.org/index.aspx?o=1026) (*e.g*., numbered containers or central telephone), clarifying whether the sequence was concealed until interventions were assigned. Numbered schools were all randomized at one time point, sequence was concealed until intervention assigned and school identity revealed . | 8 & 9 |
| Randomization -- Implementation | 10 | [Who generated the allocation sequence, who enrolled participants, and who assigned participants to their groups](http://www.consort-statement.org/index.aspx?o=1027). Trial Statistician generated sequence and assigned schools. | 8 & 9 |
| Blinding (masking) | 11 | [Whether or not participants, those administering the interventions, and those assessing the outcomes were blinded to group assignment](http://www.consort-statement.org/index.aspx?o=1028#11a). If done, [how the success of blinding was evaluated](http://www.consort-statement.org/index.aspx?o=1028#11b). Trial was not blinded. |  |
| Statistical methods | 12 | [Statistical methods used to compare groups for primary outcome(s)](http://www.consort-statement.org/index.aspx?o=1029#12a); [Methods for additional analyses](http://www.consort-statement.org/index.aspx?o=1029#12b), such as subgroup analyses and adjusted analyses. ITT analysis of abortion rates based on method of GEE | 9 |
| RESULTS Participant flow | 13 | [Flow of participants through each stage](http://www.consort-statement.org/index.aspx?o=1018) (a diagram is strongly recommended). Specifically, for each group report the numbers of participants randomly assigned, receiving intended treatment, completing the study protocol, and analyzed for the primary outcome. [Describe protocol deviations from study as planned, together with reasons](http://www.consort-statement.org/index.aspx?o=1086). | CONSORT diagram on page 21 |
| Recruitment | 14 | [Dates defining the periods of recruitment and follow-up](http://www.consort-statement.org/index.aspx?o=1087). Recruitment 1998 and 1999. Follow up to June 2005 | 6 & 7 |
| Baseline data | 15 | [Baseline demographic and clinical characteristics of each group](http://www.consort-statement.org/index.aspx?o=1088).  Well balanced between groups: 10.5% free school meals; 46.7% obtained 5 or more GCSE (Grades A*-C); 19.4% dislike school; 6.7% had sexual intercourse | Fig 3, page 27 |
| Numbers analyzed | 16 | [Number of participants (denominator) in each group included in each analysis and whether the analysis was by "intention-to-treat"](http://www.consort-statement.org/index.aspx?o=1089). State the results in absolute numbers when feasible (*e.g*., 10/20, not 50%). 113/2247 abortions control group; 119/2529 abortions intervention group | 21 and 22 |
| Outcomes and estimation | 17 | [For each primary and secondary outcome, a summary of results for each group, and the estimated effect size and its precision](http://www.consort-statement.org/index.aspx?o=1090) (*e.g.*, 95% confidence interval). | 22,23,24 |
| Ancillary analyses | 18 | [Address multiplicity by reporting any other analyses performed](http://www.consort-statement.org/index.aspx?o=1091), including subgroup analyses and adjusted analyses, indicating those pre-specified and those exploratory. |  |
| Adverse events | 19 | [All important adverse events or side effects in each intervention group](http://www.consort-statement.org/index.aspx?o=1092). |  |
| *DISCUSSION* Interpretation | 20 | [Interpretation of the results](http://www.consort-statement.org/index.aspx?o=1019), taking into account study hypotheses, sources of potential bias or imprecision and the dangers associated with multiplicity of analyses and outcomes. Compared with conventional school SRE, this form of peer-led SRE was not associated with change in teenage abortions but probably led to fewer teenage births and was popular with pupils. | 3, 15-18 |
| Generalizability | 21 | [Generalizability (external validity) of the trial findings](http://www.consort-statement.org/index.aspx?o=1094).  Schools were representative of state schools in England and peer-led intervention was designed to be generalisable to wide variety of schools | 12 and 15 |
| Overall evidence | 22 | [General interpretation of the results in the context of current evidence](http://www.consort-statement.org/index.aspx?o=1095). This form of peer-led SRE (the RIPPLE programme) merits a place within broader teenage pregnancy prevention strategies and has been recommended by Department of Health | 18 |

**www.consort-statement.org**
