# Supplementary material for: The Long-Term Effects of a Peer-Led Sex Education Programme (RIPPLE): A Cluster Randomised Trial in Schools in England
Source: PLoS Med. 2008 Nov 25;5(11):e224. doi: 10.1371/journal.pmed.0050224 (PMC2586352; doi:10.1371/journal.pmed.0050224)
Supplement: Text S2 — (2.4 MB DOC) [file pmed.0050224.sd002.doc]

**RIPPLE**

**A Randomised Intervention trial of Peer-Led Sex Education in schools in England**

**Full Protocol**

**Table of contents**

**1 Background**

**2 Study funding and organisation**

**3 Aims and objectives**

**4 Trial design**

**4.1 Follow-up**

**4.2 Trial endpoints**

**4.3 Inclusion criteria**

**4.4 Sample size**

**4.5 Method of randomisation**

**4.6 Intervention**

**-** *Training of peer educators*

*- Delivery of peer-led sex education*

**5 Plan of investigation**

**5.1 Assignment of ID codes**

**5.2 Survey methods**

**5.3 Process evaluation**

- *Focus groups*
- *Observation*
- *Interviews*

**6 Ethical issues and informed consent**

1. **Plan of analysis**

**7.1 School-level based analyses**

- 1. **Analyses based on individual pupil’s outcome**
- *Marginal models*
- *Conditional models*

**7.3 Analysis of process data**

**8 Steering committee**

**Table I Timing of research activities in relation to the intervention**

**Table II Trial timetable**

**List of Appendices (Please contact corresponding author for further information)**

Appendix 1: Parental consent letter

Appendix 2: Information sheet for year 9 pupils in intervention schools

Appendix 3: Information sheet and consent form for year 12 pupils

Appendix 4: Instructions for conduct of questionnaire survey

Appendix 5: Guide for interviews with teachers

Appendix 6: Guide for focus group discussions with Year 9 pupils

**A Randomised Intervention trial of Peer-Led Sex Education in schools in England (RIPPLE)**

## **1. Background**

###### *The need to improve the sexual health of young people*

## Sexual health is integral to the health of the public and includes the prevention of sexually transmitted infections (STI) and unintended pregnancy. Important consequences of STI include pelvic inflammatory disease, infertility, cervical cancer and increased susceptibility to HIV infection. Unintended pregnancy is associated with poorer health, economic and social outcomes for both mother and child [1].

## The impetus for promoting sexual health among young people in the UK has been generated by concerns over high rates of teenage conceptions – the UK has the highest rate in Europe – and the need to control the spread of STI including HIV. National survey data indicate that first sexual intercourse is occurring at an increasingly early age [2]. The younger a person is at first sexual intercourse, the less likely it is that contraception will be used. Half of young people who are sexually active before age 16 use no contraception and half of the pregnancies in this age group end in abortion. Despite government targeting of sexual health as a key area for public health action in the UK, recent national data show worsening trends and substantial inequalities in teenage STI and pregnancy [3-4]. The UK government has recently announced a national campaign to tackle the problem of teenage pregnancy [4]. The campaign goal is to halve the number of conceptions in under 18 year olds by 2010.

*How effective is school sex education in tackling this problem?*

The great majority of young people receive some kind of sex education in school^5^ and the government recognises school sex education as an important strategy to promote young people’s sexual health and reduce teenage pregnancy. However, there is little systematic information about the extent to which sex education influences behaviour even though schools are the setting for much sexual health promotion work with young people. There is also wide debate about the merits of school sex education, and in parcticular whether sex education reduces or increases sexual risk behaviour [5].

Current theories of sexual health promotion emphasise concepts of empowerment, participation and mobilisation of groups and individuals to define and meet their own health needs [6]. Research into young people’s own experiences and perspectives questions the appropriateness of traditional didactic methods of teaching sex education in schools. Young people are often critical of the content and style of the sex education they receive at school. Teachers may be perceived as embarrassed and uneasy about teaching sex education [7,8]. Young people commonly report that coverage of the subjects they wanted to know about is inadequate, while subjects with which they were already familiar received too much attention [4].

*Research into the effectiveness of sexual health interventions for young people.*

Uncertainty about the effectiveness of different methods of sex education reflects a lack of scientific evaluation. The background to RIPPLE is a systematic review of sexual health promotion interventions for young people [9]. The review found 65 separate studies evaluating the outcome of interventions, of which only 12 (18%) were methodologically sound. Criteria used in evaluating the studies were based on the use of a control group, pre and post intervention measures and reporting on all outcomes included in the aims of the study. Eleven of the methodologically sound interventions were carried out in North America and one in Finland. Only two of the sound evaluations (both in North America) were able to show an impact on self-reported sexual behaviour. The review recommended that evaluation of sexual health interventions should incorporate experimental designs (randomisation), adequate sample sizes and the long term follow-up of participants.

A more recent systematic review [10] compared the results of randomised trials with observational studies of interventions to prevent adolescent pregnancy. Thirteen trials and 17 observational studies were identified. Based on four comparable outcomes (sexual intercourse, contraceptive use, responsible sexual behaviour and pregnancy), the review found that the observational studies yielded systematically greater estimates of intervention effects than randomised trials. It appeared unlikely that the interventions evaluated in the observational studies were inherently more effective than those studied in randomised trials. A more likely explanation for the findings is that adolescents assigned to the intervention in the observational studies were destined to have better outcomes than control adolescents irrespective of the intervention. In effect, the studies were probably comparing different populations rather than different interventions. The review concluded that, wherever possible, recommendations and public policy should be based on randomised trials.

*Peer-led sex education*

Peer-led health interventions are currently enjoying a wave of popularity and in July 1999 the Social Exclusion Unit report on teenage pregnancy identified peer-led sex education as a possible prevention strategy [4]. The term “peer” relates to one of equal status. Peer-led (sex) education can therefore be defined as “teaching or sharing of (sexual health) information, values and behaviours by members of similar age or status group.” [11] A number of psychosocial theories of behaviour change have been employed to provide a framework for understanding the processes involved peer led education including social learning theory [12], social inoculation theory [13] and diffusions of innovations theory [14] (see reference [15] for overview). Common to all these theories is the idea that behaviour is influenced by an individual’s social network and the normative values and beliefs that exist among friends, peers and family. Peer led programmes claim an advantage over traditional teacher-led methods through the belief that peer leaders act as positive role models with whom the target group can relate, conveying information in a way that appeals to the target group. The egalitarian nature of the interaction between young people provides the opportunity to communicate openly and discuss sexual health issues in ways that have specific social and cultural relevance. Peer leaders are viewed as credible sources of health promoting information. Such characteristics facilitate a process whereby young people acquire necessary information on sexual health and explore attitudes within a context which directly relates to their own experience of their social environment.

The rising popularity of peer education has brought the methods under particular scrutiny. A recent review of peer delivered health interventions for young people funded by the Department of Health found some evidence to support the effectiveness of peer delivered interventions but commented that the variability of different peer interventions and the scarcity of methodologically sound studies made it difficult to draw conclusions about the specific characteristics of effective peer delivered interventions. [16] In most reports the relationship between theory and intervention strategies was unclear. Studies exploring the implementation of peer led methods tended to focus on the acceptability of the intervention. Very few examined the relationship between peer leaders and their target group. Furthermore, very few studies have integrated both process and outcome evaluations. Other reviews of peer-led education have reached more negative conclusions and challenged assumptions that peer leaders are perceived as credible sources by the target group, [17] or that they model behaviours and attitudes that are necessarily compatible with the agendas of health professionals.

*Rationale for a large randomised controlled trial of peer-led sex education*

Randomised trials are recognised as the gold standard for evaluating health care interventions. Although evaluation of complex interventions, such as those aiming to change sexual behaviour, presents particular challenges, [18] the systematic comparison, described above, between trials and observational studies in adolescent pregnancy prevention confirms the need for randomised trials to strengthen the evidence base in this field of health promotion. [10] Although peer-led sex education is popular with young people, its effectiveness, in terms of health outcomes, is unknown. The use of outcome measures such as abortion rates requires a trial of sufficient sample size to detect a measurable difference in these rates. In 1993 the abortion rate in England and Wales was 22 per 1000 in women aged 16-19 years. To detect a difference of one third reduction in this rate would result in only seven fewer pregnancies per 1000 women. It is therefore important to have a study that is large enough to detect change that can be attributed to the intervention rather than chance variations in abortion rates. STD rates peak at ages 16-19 in women and later in men. Using STDs as outcome measures of an intervention implemented before 16 requires follow-up to the age of 20. Other important outcomes that occur more commonly, or can be collected more easily, include condom use at first intercourse, contraceptive use, regretted sexual encounters, knowledge, attitudes and intentional and actual sexual behaviour.

There is general agreement that effective sex education should not be measured purely in terms of health outcomes, but should also include measures of the quality of sexual relationships and perhaps the wider impact of the intervention in schools. Pupil satisfaction with sex education and their views on the appropriateness of what they receive should also receive attention. In addition, evaluation of the processes involved in implementing an intervention should be an integral part of any well-designed trial of a complex intervention. The process evaluation is key to explaining the observed outcomes and to the future implementation of a successful intervention.

*A pilot study*

The Department of Health funded a 15month pilot study for this trial that was conducted in four schools in the London area and completed in February 1997. The aim was to assess the feasibility and acceptability of conducting a large RCT, to pilot the intervention and the study methods, including baseline and post-intervention questionnaires. [19] The intervention followed a model developed by the Ibis Trust, an independent organisation with expertise in developing and implementing peer-led education programmes. The pilot study showed that the intervention needed to be modified to produce a more standardised intervention that could be evaluated in a large RCT. The pilot showed that the evaluation methods were feasible and acceptable to pupils, parents, staff and governors. [19]

In summary, peer-led sex education is clearly popular with staff and pupils, but its effectiveness, in terms of sexual health outcomes, is unknown. RIPPLE (A Randomised Intervention trial of Peer-Led Sex Education in schools in England) is a school-based randomised controlled trial in southern England designed to provide more reliable evidence about the long term impact of peer-led sex education on sexual health.

2. Trial funding, investigators, organisation and management

The trial is funded by the Medical Research Council (MRC) for five years from August 1^st^ 1997 (Phase I), with four years additional funding for long term follow-up to ages 19-20 (Phase II) dependent on the results of Phase I.

The trial is run jointly between two academic institutions, the Department of Sexually Transmitted Diseases, at UCL and the Social Science Research Unit at the Institute of Education, and an external group of practitioners with expertise in peer-led education. The trial is therefore multidisciplinary with collaboration between clinical epidemiologists and social scientists. The Department of STD acts as the data management site and the MRC grant is administered through the Institute of Education.

The research team comprises project directors, research fellows, data managers, statisticians and an external group of peer trainers, plus administrative support. The project directors are Dr. Judith Stephenson (UCL, who has responsibility for overall management of the trial), Professor Ann Oakley (IoE, who has responsibility for the process evaluation), Professor Anne Johnson (UCL, who advises on aspects on methodology) and Angela Flux who has responsibility for training peer-educators and implementing the peer-led intervention in schools. Other key members of the research team are the research fellows (Simon Forrest, Vicki Strange and Susan Charleston) who carry out the surveys, fieldwork and interviews in schools, the data managers (Gayle Johnston / Sarah Hambidge) and statisticians (Stephanie Black / Ann Petruckevitch, supervised by Professor Abdel Babiker (MRC Clinical Trials Unit). A commercial data management company enters the questionnaire data onto computer. The trial has a steering committee (page 19). The usual indemnities for university researchers apply to RIPPLE.

**3. Aims and objectives**

The aim of the trial is to evaluate the effectiveness of school-based peer-led sex education in promoting young people’s sexual health

The primary objective is to measure the effectiveness of peer-led sex education in reducing the incidence of unwanted pregnancy, termination of pregnancy, unprotected sexual intercourse and STI, and in improving the quality of sexual relationships. Secondary objectives relate to the wider effects on school ethos, particularly on the status and implementation of sex education in schools, and on relationships between staff and students.

4. Trial design

RIPPLE is an open, stratified, cluster randomised controlled trial with extensive process evaluation as well as outcome evaluation. Intervention occurs at ages 13-14 years. In Phase 1 of the trial, students are followed-up to age 15-16; in Phase 2 follow-up of both arms is extended to ages 19 –20.

- 1. **Follow-up**

In Phase I, the first follow-up is conducted 6 months post-intervention at ages 14-15; and the second follow-up is conducted 18 months post-intervention at ages 15-16. At each follow-up information is collected by pen and paper questionnaire completed in the classroom setting. For pupils absent on the day of the survey, a questionnaire is left with the contact teacher and each absentee is invited to complete the questionnaire as soon as they return to school.

In Phase II , the third follow-up is conducted in the community at ages 17-18; the fourth and final follow-up is conducted in the community at ages 19-20. At this stage, follow-up information is collected through 1) anonymised linkage to statutory abortion and birth notifications, 2) postal questionnaire and 3) postal saliva or urine samples which will be tested for markers of STI exposure (see trial endpoints).

##### Trial endpoints

**Phase 1**: Primary endpoints are unprotected sexual intercourse before the age of 16 and regretted sexual relationships. Other phase 1 endpoints relate to the Government’s Teenage Pregnancy Strategy, [3] including pupil satisfaction with school sex education, ability to access sexual health services, condom use at first and last intercourse and appropriate use of contraception. In terms of secondary objectives, the process evaluation will identify reasons for continuing, extending or changing the method of sex education used during the study, and assess staff attitudes to future provision of sex education.

**Phase II**: Primary endpoint is cumulative incidence of termination of pregnancy by age 19-20. The Chief Medical Officer has agreed to the use of statutory abortion notifications for this purpose. Anonymised data will be obtained from the Office of National Statistics on abortions in both experimental and control schools. This means that there will be no loss to follow-up for the primary outcome. In addition to unprotected intercourse and regretted sexual relationships, phase II outcomes will include markers of exposure to STI i.e chlamydia and herpes genital infections through measurement of type specific *C. trachomatis* and Herpes simplex virus 2 antibodies respectively.

### 4.3 Inclusion and exclusion criteria

Schools in southern England are eligible to take part in the trial if they are comprehensive, mixed sex, non-selective and take pupils until age 18. Schools that are already implementing peer-led sex education are not eligible (but schools that use peer-led methods in other areas e.g. anti-bullying programmes are eligible). In each school, all pupils in Year 9 (13-14 year olds) are eligible for the study unless their parents have withdrawn them from the research study. In experimental schools, all pupils in Year 12 (aged 16-17 years) are eligible to be peer-educators, and no one who volunteers is excluded.

**4.4 Sample size and power calculations**:

The power calculation takes into account that the school, not the individual, is the unit of randomisation. [20] The number of schools required for the trial depends on 1) the expected incidence rate of the main outcome in the absence of any intervention 2) the variability in the incidence rate between schools 3) the number of participating pupils in each school 4) the magnitude of effect of the intervention that the trial is intended to detect and 5) the desired degree of certainty (statistical power) with which such an effect is to be detected. Termination of pregnancy was chosen as the primary outcome on which to estimate the size of the trial because it is an important and relatively uncommon event for which reliable data are available. Based on the legal abortion rate for 16-19 year olds in England and Wales in 1994 (23 per 1000), the expected cumulative incidence of termination by age 20 will be at least 9%. The variability in outcome between schools can be expressed as k (the coefficient of variation (SD/Mean) of the incidence rate) which, in the absence of reliable data, was assumed to be 0.2. The same variability can also be expressed as a standard deviation of 1.8% which translates into nearly 3 fold variation between schools in the abortion rate from 5% to 13%. With these assumptions, 14 schools, with an average of 150 girls in each, would be needed in each arm to detect a one third reduction in the termination rate, from 9% in control schools to 6% in intervention schools, with over 80% statistical power at 5% significance. However, the value of k, or the extent of between school variability can be reduced by effective stratification of schools (see 4.5 below), thus making the sample size estimates conservative.

The trial will have over 90% power to detect a reduction in the proportion of pupils having unprotected intercourse before age 16, from 35% to 25%, assuming a standard deviation of 8.2% which gives a range of 19% to 51% between schools. Two successive cohorts of Year 9 pupils (with average Year size 150 pupils, or 75 girls) are recruited in each school to provide adequate statistical power. Total number of participants is over 8000.

**4.5 Method of randomisation**.

The method is stratified randomisation with the school as the unit of randomisation. Before randomisation, 28 eligible schools are ranked according to a composite score developed to assess risk status. The score is based on seven factors:

1. socioeconomic status (the proportion of pupils having free school meals),
2. ethnicity (the proportion of Black and Asian students),
3. educational attainment (the proportion gaining 5 or more GCSEs),
4. continuing education (the proportion staying on after age 16),
5. the quality and quantity of current school sex education,
6. the attitude of the school towards health promotion (the availability of information and links with outside agencies, and
7. local family planning services (convenient location or opening times, youth-friendliness and level of use by students).

None of the factors is weighted with respect to the others. Information about factors 1-4 is obtained directly from school staff or from recent Ofsted reports; information about factors 5-7 is obtained directly from school staff by interview or questionnaire. After ranking by composite score, the 28 schools are divided into 3 equal sized strata of low, medium and high risk schools. Within each stratum, schools are randomised to experimental (peer-led sex education) or control (usual practice) arm, giving a total of 14 experimental and 14 control schools.

**4.6 The intervention**

*The training of peer educators*

Peer educators receive a standard training programme that is delivered by an external team of peer trainers with expertise in sexual health promotion. The training takes place in the Spring term (and the delivery of the programme in the following Summer term). All male and female Year 12 students (16/17 years old) in each experimental school are eligible to volunteer to undertake this training and participants receive accreditation from the University of London acknowledging their involvement in the programme. The training programme comprises a series of weekly meetings in which the peer educators are given information about sexual health issues and the opportunity to experience and develop participatory learning strategies and activities (i.e. role-play, use of quizzes, games, managing discussions, etc.). Peer educator training culminates in a workshop over two consecutive days which focuses on knowledge about relationships, contraception and sexually transmitted infections and techniques of classroom management and group facilitation. Peer educators either collect or are provided with information about local sexual health services. Emphasis is placed on training peer educators through techniques which they can adopt in the intervention. The aim is develop classroom strategies, appropriate to the age and experience of students in the intervention group, through which their communication and negotiation skills in relation to sexual behaviour and relationships will be enhanced. The content and delivery of the peer educator training is standardised as far as possible across the experimental schools. Training meetings are observed by members of the researcher team and peer educators participate in a questionnaire survey prior to the training. This survey is repeated after delivery of the intervention and focus group discussions undertaken with the peer educators .

##### *The delivery of the peer-led sex education programme*

The peer-led sex education programme is delivered in the Summer Term. It comprises three classroom sessions each lasting a minimum of one hour. The sessions focus on relationships, contraception and sexually transmitted infections, in order to match the objectives of the intervention to the endpoints of the trial (see above). Sessions may be timetabled in a variety of subject areas in accordance with the usual school practice for sex education provision. They are delivered by mixed sex teams of peer educators who deliver all three sessions to one group of students from Year 9. Teachers are not in attendance in the classroom during the sessions. Peer educators adopt a less formal role and approach than teachers and make more use of participatory classroom teaching techniques. These involve games and small groupwork, discussions, brainstorms, role-playing and demonstrating how to use condoms. The approach emphasises development of skills for sexual negotiation as well as knowledge about pregnancy, contraception, sexually transmitted diseases and the use of sexual health and contraceptive services. The organisation of the intervention their content and delivery is standardised as far as possible across the experimental schools.

**Table I Timing of research activities in relation to the intervention**

| Research Activities | | Intervention | |
| --- | --- | --- | --- |
|  |  | Experimental schools | Control schools |
| Autumn 1997 | - **Baseline questionnaire to yr9** |  |  |
| Spring  1998 | - Observation of recruitment - Baseline questionnaire with all peer educators - **Interviews with teachers** | Recruitment of peer educators | Continue with sex education as planned |
| Summer 1998 | - Observation of pre training and training sessions - **Observation of sex education sessions delivered to yr9*** - Follow up questionnaire with all peer educators - **Focus groups with yr9 students** - Focus groups with peer educators | Preparatory sessions with peer educators  Needs assessment by peer educators with yr9.  Training of peer educators  Delivery of sex education classes | Continue with sex education as planned |
| Autumn 1998 | - **Baseline questionnaire to yr9** - **Follow up questionnaire to yr10** |  |  |
| Spring  1999 | Repeat all of above for cohort 2 | | |
| Summer 1999 |  |  |  |
| Autumn 1999 | - **2^nd^ follow questionnaire to yr11** - **Follow up questionnaire to yr10** |  |  |

* sex education in control schools is observed through out the year and depends on the timing of delivery which is decided by individual schools.

**Note: All activities in bold were carried out in both experimental and control schools. Others were carried out only in experimental schools**.

**5 Plan of investigation**

**5.1 Trial register and assignment of ID codes**

The trial register is composed of all pupils enrolled in year 9 at the time of the baseline survey. Each pupil on the trial register is assigned a unique ID code, composed of 8 characters. The ID code consists of a two figure code for the school (01 to 28) a single digit for the Year 9 cohort (1 or 2) a three figure code for the pupil (001 to 900) and a check letter at the end.

**5.2** **Survey Methods**

In phase I, questionnaire surveys are carried out in the classroom at baseline (pre-intervention), at 6 and 18 months post intervention for the two successive Year 9 cohorts. The questionnaires include measures of knowledge, attitudes, self-efficacy, sexual behaviour including vaginal intercourse, condom and contraceptive use, and views on any previous sex education. The questionnaires were developed and piloted in consultation with students, and include space for comments. Where possible, a member of the research team is present to explain the purpose of the questionnaire, reassure students about the confidential nature of their responses and answer any queries. In phase II, postal questionnaires, together with a urine or saliva “home testing kit” will be sent to all pupils on the trial register who give consent to be approached by the research team after they have left school.

**5.3 Process evaluation**

Extensive process evaluation is an integral part of the research design. The primary aims of this are: to document how the intervention is implemented in the experimental schools, and what kind of sex education is provided in control schools; to collect information from all the study schools about the experience and impact of taking part in the RIPPLE study; to examine the extent to which sex education in control schools may have been 'contaminated' by knowledge about the peer-led programme and to collect data on the key processes involved in the provision and receipt of peer-led sex education which can then help to interpret outcome data. Information is gathered about all key events in the implementation of the peer-led intervention in all experimental schools. Information about the sex education curriculum is collected from all control schools, while observation and focus groups are carried out in a 50% random sample of control schools. In addition, information is colllected about the school environments in which the trial is taking place. For example, the quality of relationships between staff and students and the status of the school within the community are assessed. Some process information is gathered within the questionnaire survey. However most of it is obtained by using other methods, including focus groups, interviews with staff and observations of peer educator training and delivery of the intervention.

*Focus groups*

Focus groups are carried out with single sex group of Year 9 students following the peer-led intervention in experimental schools and following teacher-led sex education in control schools. Focus groups are also undertaken with mixed sex groups of peer educators in the experimental schools. The aim of the focus groups is to gain insight into students’ perceptions and evaluation of sex education i.e. what they liked and disliked, why they thought parts of the sessions were successful or not, how they perceived the peer-educators or teachers as providers of sex education, and their views on wider issues about the school environment and culture. For example, the ways in which staff treat students, the relationship between girls and boys, and the availability and accessibility of welfare support to students.

*Observation*

Key processes in the implementation of the peer-led intervention are observed in all the experimental schools and teacher led sex education is observed in half of the control schools (randomly selected). Observation in the experimental schools includes training meetings with peer educators, in and outside school, and a sample of the peer led sessions with the Year 9 students in each school. For each event, a full description (narrative) is written by the researcher including who was present, what was said, the activities undertaken, interactions between participants, the atmosphere, and the occurrence of significant events such as pupils being dismissed from classrooms. The aim is to gather detailed data on what information was provided, the way in which peers/ teachers delivered the sessions and interacted with the Year 9 students, students' responses, the type of language used, and how the peer-led intervention was implemented.

*Interviews with staff*

Interviews are carried out with staff responsible for sex education and Headteachers in all schools. The aim is to gather information about the content and delivery of sex education, and teachers’ evaluation of it. An additional aim is to identify input from other health-related organisations and services, the extent of teacher training and needs, and how sex education fits within the school curriculum. Information is also gathered about school ethos and the wider context in which sex education is provided. The National Healthy Schools Standard [21] provide a framework for this, highlighting good practice as that which involves parents and carers, the appropriate training and support of school staff and ensuring that the views of the pupils are listened to.

**6 Ethical issues and informed consent**

Consent is sought from parents and Year 9 pupils for participation in the research aspects of RIPPLE, not for the intervention *per se.* This is appropriate because schools can choose to provide peer-led sex education, or not, in the absence of any research study, and parents can choose to withdraw their child from any form of school sex education. The method is as follows: All parents of pupils in Year 9 are sent a letter informing them about the study and asking for their consent. Individual schools can decide whether parental consent will be opt-in (active, signed consent), or opt-out (in which case no expressed objection was interpreted as consent). Year 9 pupils are also given written and oral information about the study before being invited to fill in questionnaires. The researchers explain to pupils that they do not have to fill in the questionnaire if they do not want to. In experimental schools, signed consent is sought from the peer-educators themselves. Consent to take part in RIPPLE therefore amounts to consent for 13-14 year old pupils in all schools to take part in research activities (e.g. filling in questionnaires and taking part in focus groups) and consent from 16-17 year old pupils in the experimental schools to train as peer-educators, deliver sessions to Year 9 pupils and take part in research activities. The study was approved by the UCL/UCLH joint committees on the ethics of human research. School staff and students will receive annual feedback on the progress of the study through a RIPPLE newsletter.

At the time of the 18 month post intervention follow-up, all pupils are asked whether the research team can approach them after they have left school and invite them to continue their participation in the trial. They are asked to indicate their consent by filling in their name and address. Further ethics committee approval will be sought for phase II of the study because it will involve testing for exposure to sexually transmitted infections.

### Plan of analysis

For each of the two primary end points (unprotected sexual intercourse before the age of 16 and termination of pregnancy), the following analyses will be undertaken:

**7.1 School‑level based analyses**

The average annual incidence rates (total number of events divided by total person‑years) in the intervention (R_0_) and the control (R_1_) schools will be compared. The observed difference R_0_ - R_1_ will be referred to a normal distribution with mean $\lambda_{0}-\lambda_{1}$ and variance $\sigma_{0}^{2}+\sigma_{1}^{2}$; where $\lambda_{i}$ is the true mean annual incidence rate in group i ( i=0,1),$\sigma_{i}^{2}=\lambda_{i}/Y_{i.}+k^{2}\lambda_{i}^{2}(\sum_{j} Y_{ij}^{2})/(Y_{i.})^{2}$, $Y_{ij}$ is the person years in school j of arm i, $Y_{i.}$ is the total person-years in arm i and k^2^ is the coefficient of variation between schools in the annual incidence rate. This analysis will be undertaken both unstratified and stratified by the three strata used in the randomisation.

**7.2 Analyses based on individual pupil’s outcome**

*Marginal models:*

The marginal probabilty of unprotected intercourse by age 16 and of termination of pregnancy by age 19 will be compared between the intervention and control schools using the Generalized Estimating Equations approach of Liang and Zeger. [22]

*Conditional Models:*

The probability of outcome conditional on school effect will be compared between the intervention and control arms by fitting multilevel methods with random effects for school and cohort using MLwin.

Secondary analyses will adjust the comparisons using marginal models and conditional models for pupil level covariates: sex (first primary endpoint only), ethnic origin, social class etc.

- 1. **Analysis of process data**

Data collected from focus group discussions with students and peer educators, interviews with teachers, observation of lessons and researcher field diaries are transcribed and analysed using a variety of approaches including thematic content analysis and discourse analysis. A particular challenge is to move beyond the usual practice in which 'qualitative' data are analysed separately and sit alongside 'quantitative' measures of outcome. To integrate process and outcome evaluations, qualitative process data are coded to create variables which describe the implementation and acceptability of the intervention. Data on these variables are entered into the computer database and analysed with the data from questionnaire surveys. Data from the process evaluation may also be analysed independently of the data from the questionnaire surveys to generate hypotheses about the importance and impact of local effects on outcomes in individual or groups of schools. The data will also be analysed to address other important questions, for example to describe the type of young people who volunteer to be peer educators and the impact on them of their involvement in the programme.

8 Trial Steering Committee

The trial has a steering committee whose members are:

Professor Peter Smith (Chair), LSHTM

Mr John Holman, Head of Watford Grammar School

Ms Gill Morris, Health Education Co-ordinator, Camden Education Department

Dr. Sandra Williams, R&D Division, Department of Health

Professor Graham Hart, MRC Sociology Unit

MRC Observer.

The steering committee provides advice to the research team, and ensures that the trial is conducted to the standards set out in the MRC Guidelines for Good Clinical Practice. In particular, the steering committee is asked to concentrate on progress of the trial, adherence to the protocol, participant safety and consideration of new information. It meets approximately twice a year and provides an annual report to the MRC on the progress of the trial. At each meeting, the research team presents at least as much information about the status / progress of the trial as the steering committee needs to make its report. These meetings also provide an opportunity for members of the research team and steering committee to discuss and anticipate potential problems including relations with the media. An independent data-monitoring committee was considered unnecessary by the MRC.

**Reference List**

1. Dickson, R., Fullerton, D., Eastwood, A., Sheldon, T., and Sharp, F. Preventing and reducing the adverse effects of unintended teenage pregnancies. Effective Health Care 3(1), 1-12. 1997.

2. Johnson AM, Wadsworth J, Wellings K, Field J. Sexual Attitudes and Lifestyles. Oxford: Blackwell Scientific Press, 1994.

3. Nicoll A, Catchpole M, Cliffe S, Hughes G, Simms I, Thomas D. Sexual health of teenagers in England and Wales: analysis of national data. *Br.Med.J.* 1999;**318**:1321-3.

1. Social Exclusion Unit. Teenage pregnancy. 1-139. July 1999. Department of Health.
2. Dean M. Muddle over sex education. *Lancet* 1994;**343**:1149.

6. Abraham C,.Sheeran P. Modelling and modifying young heterosexuals' HIV-preventive behaviour; a review of theories, findings and educational implications. *Patient Education and Counseling* 1994;**23**:173-86.

7. Kirby D. Sex and HIV/AIDS education in schools. *Br.Med.J.* 1995;**311**:403-.

8. Coleman LM,.Ford NJ. An extensive literature review of the evaluation of HIV prevention programmes. *Health Education Research* 1996;**11**:327-38.

9. Oakley A, Fullerton D, Holland J, Arnold S, France-Dawson M, Kelley P *et al*. Sexual health education interventions for young people: a methodological review. *Br.Med.J.* 1995;**310**:158-62.

10. Guyatt GH, DiCenso A, Farewell V, Willan A, Griffith L. Randomized trial versus observational studies in adolescent pregnancy prevention. *J.Clin.Epidemiol.* 2000;**53**:167-74.

11. Sciacca JP. Student peer health education: a powerful yet inexpensive helping strategy. *The Peer Facilitator Quarterly* 1987;**5**:4-6.

12. Bandura A. Social learning theory. New Jersey: Prentice-Hall, 1977.

13. McGuire WJ. The nature of attitudes and attitude change. In Lindzey G, Aronson E, eds. pp 136-314. Addison-Wesley, 1968.

14. Rogers EM. Diffusions of innovations. New York: Free Press, 1983.

15. Turner G,.Shepherd J. A method in search of a theory: peer education and health promotion. *Health Education Research* 1999;**14**:235-47.

1. Harden, A., Weston, R., and Oakley, A. A review of the effectiveness and appropriateness of peer-delivered health promotion interventions for young people. 1999. London, EPI-Centre.
2. Milburn K. A critical review of peer education with young people with special reference to sexual health. *Health Education Research* 1995;**10**:407-20.

18. Stephenson J,.Imrie J. Why do we need randomised controlled trials to assess behavioural interventions? *Br.Med.J.* 1998;**316**:611-3.

19. Stephenson JM, Oakley A, Charleston S, Brodala A, Fenton K, Petruckevitch A *et al*. Behavioural intervention trials for HIV/STD prevention in schools: are they feasible? *Sexually Transmitted Infections* 1998;**74**:405-8.

20. Hayes R, Mosha F, Nicoll A, Grosskurth H, Newell J, Todd J *et al*. A community trial of the impact of improved sexually transmitted disease treatment on the HIV epidemic in rural Tanzania. 1. Design. *AIDS* 1995;**9**:919-26.

1. Rivers, K., Aggleton, P., Chase, E., Downies, A., Mulvihill, C., Sinkler, P., Tyrer, P., and Warwick, I. Setting the standard: Research linked to the development of the National Healthy School Standard (NHSS). Department of Health and Department of Education and Employment. 2000. London.
2. Liang K-Y and Zeger SL, ‘Longitudinal data analysis using generalized linear models’. Biometrika, 1986;**73:** 13-22.

# Table II - TRIAL TIMETABLE

|  | **First cohort** | |  | |  |
| --- | --- | --- | --- | --- | --- |
|  | **Experimental schools** | **Control schools** |  | |  |
| Autumn term 1997 | 1^st^ questionnaire to Yr 9 pupils | 1^st^ questionnaire to Yr 9 pupils |  |  | Stages already completed |
| **Spring term 1997** | Recruitment of peer volunteers | Continue with sex education as planned |  |  |  |
| **Summer term 1998** | Training of peer educators and delivery of sex education classes to Yr 9 pupils |  | Second cohort | | |
|  |  |  | Experimental schools | | Control schools |
| **Autumn term 1998** | 2^nd^ questionnaires to Yr 10 pupils | 2^nd^ questionnaire to Yr 10 pupils | **1^st^ questionnaire to Yr 9 pupils** | | 1^st^ questionnaire to Yr 9 pupils |
| **Spring term 1998** |  |  | Recruitment of peer volunteers | | Continue with sex education as planned |
| **Summer term 1999** |  |  | Training of peer educators and delivery of sex education classes to Yr 9 pupils | |  |
| **Autumn term 1999** |  |  | 2^nd^ questionnaire to Yr 10 pupils | | 2^nd^ questionnaire to Yr 10 pupils |
| **Spring term 2000** | 3^rd^ questionnaire to Yr 11 pupils | 3^rd^ questionnaire to Yr 11 pupils |  | |  |
| **Spring term 2001** |  |  | 3^rd^ questionnaire to Yr 11 pupils | | 3^rd^ questionnaire to Yr 11 pupils |
| **Spring 2002** | 4^th^ questionnaire in the community with postal specimens for STI testing | |  | | |
| **Spring 2003** |  | | 4^th^ questionnaire in the community with postal specimens for STI testing | | |
| **Spring 2004** | Final questionnaire in the community with postal specimens for STI testing | |  | | |
| **Spring 2005** |  | | Final questionnaire in the community with postal specimens for STI testing | | |
| **Summer 2005** | Anonymised linking with routine data on conceptions and abortions. Data analysis and report writing | | | | |
